# Exosomal PGAM1 promotes prostate cancer angiogenesis and metastasis by interacting with ACTG1

**DOI:** 10.1038/s41419-023-06007-4

**Published:** 2023-08-04

**Authors:** Jun-qi Luo, Tao-wei Yang, Jun Wu, Hou-hua Lai, Li-bin Zou, Wen-bin Chen, Xu-min Zhou, Dao-jun Lv, Sheng-ren Cen, Zi-ning Long, Yi-you Mao, Peng-xiang Zheng, Xiao-hong Su, Zhi-yong Xian, Fang-peng Shu, Xiang-ming Mao

**Affiliations:** 1 Department of Urology, Zhujiang Hospital, Southern Medical University, Guangzhou, Guangdong China; 2 Department of Urology, The Third Affiliated Hospital of Guangzhou Medical University, Guangzhou, Guangdong China; 3 Department of Urology, Guangdong Provincial People’s Hospital’s Nanhai Hospital, 23 Pingzhouxiadong Road, Foshan, 528251 China; 4 Department of Urology, Guangzhou Women and Children’s Medical Center, National Children’s Medical Center for South Central Region, Guangzhou Medical University, Guangzhou, Guangdong China

**Keywords:** Tumour angiogenesis, Metastasis

## Abstract

Tumor-derived exosomes and their contents promote cancer metastasis. Phosphoglycerate mutase 1 (PGAM1) is involved in various cancer-related processes. Nevertheless, the underlying mechanism of exosomal PGAM1 in prostate cancer (PCa) metastasis remains unclear. In this study, we performed in vitro and in vivo to determine the functions of exosomal PGAM1 in the angiogenesis of patients with metastatic PCa. We performed Glutathione-S-transferase pulldown, co-immunoprecipitation, western blotting and gelatin degradation assays to determine the pathway mediating the effect of exosomal PGAM1 in PCa. Our results revealed a significant increase in exosomal PGAM1 levels in the plasma of patients with metastatic PCa compared to patients with non-metastatic PCa. Furthermore, PGAM1 was a key factor initiating PCa cell metastasis by promoting invadopodia formation and could be conveyed by exosomes from PCa cells to human umbilical vein endothelial cells (HUVECs). In addition, exosomal PGAM1 could bind to γ-actin (ACTG1), which promotes podosome formation and neovascular sprouting in HUVECs. In vivo results revealed exosomal PGAM1 enhanced lung metastasis in nude mice injected with PCa cells via the tail vein. In summary, exosomal PGAM1 promotes angiogenesis and could be used as a liquid biopsy marker for PCa metastasis.

## Introduction

The tumor microenvironment (TME) plays a crucial role in cancer metastasis [1]. Tumor metastasis is a highly coordinated, dynamic multi-step process involving invasion, extravasation, migration, and angiogenesis [2]. In the TME, angiogenesis plays a significant role in cancer metastasis [1] and podosome formation, a key component of neovascularization [1, 3, 4]. However, studies have identified only a few TME-related biomarkers for the diagnosis and prognosis of patients with cancer [5, 6]. Globally, prostate cancer (PCa) is the most prevalent cancer in men endangering their health [7]. Furthermore, angiogenesis and metastasis are the leading cause of PCa-related deaths [8, 9]. Therefore, there is an urgent need to identify PCa progression-related biomarkers [10].

Cell-cell communication aids cells in adjusting to changes in intra- and extracellular environments [11]. Exosomes are extracellular vesicles with a 40–150 nm diameter and contain proteins, lipids, and nucleic acids [12]. Exosomes and their contents mediate signaling and exchange between cells [13, 14]. Mounting evidence suggests cells or plasma-derived exosomes promote angiogenesis in several cancers [6]. Furthermore, exosomes derived from the plasma of patients with cancer can serve as reliable markers for cancer diagnosis [15]. However, the mechanism underlying angiogenesis and cancer metastasis mediated by tumor-derived exosomes is unclear and should be investigated further.

Phosphoglycerate mutase 1 (PGAM1) is a key enzyme involved in aerobic glycolysis that catalyzes the conversion of 3-phosphoglycerate (PG) to 2-PG [16]. Previous studies have shown the involvement of the glycolytic enzymatic activity of PGAM1 in cell proliferation, whereas the non-metabolic activity of PGAM1 plays a role in the migration and invasion of cancer cells [17, 18]. In addition, studies have shown the involvement of PGAM1 in cancer progression. Moreover, in PCa, PGAM1 secreted by exosomes induces cancer progression [19–22]. However, the correlation between exosomal PGAM1 and PCa metastasis is still unclear.

ACTG1 encodes for the cytoskeletal protein γ-actin, which plays a role in non-muscle cells. γ-actin is an essential component of the cell migration machinery involved in rearranging dynamic cytoskeletal networks. Studies have shown the involvement of γ-actin in the onset and progression of several diseases like cancer, brain malformations, and hearing loss [23]. However, there is a lack of research on ACTG1 and PCa metastasis.

Our results revealed an increase in exosomal PGAM1 levels in the plasma of patients with metastatic PCa compared to patients with non-metastatic PCa and healthy controls. Further, an increase in the angiogenic and proliferative capacities of human umbilical vein endothelial cells (HUVECs) treated with exosomal PGAM1 was observed. Next, we evaluated the correlation between PGAM1 and ACTG1 to determine the underlying mechanism of angiogenesis in PCa. Together, our results revealed that exosomal PGAM1 could be a novel biomarker for diagnoses of patients with metastatic Pca.

## Results

### Correlation between high exosomal PGAM1 levels and PCa metastasis

**Fig. 1:**
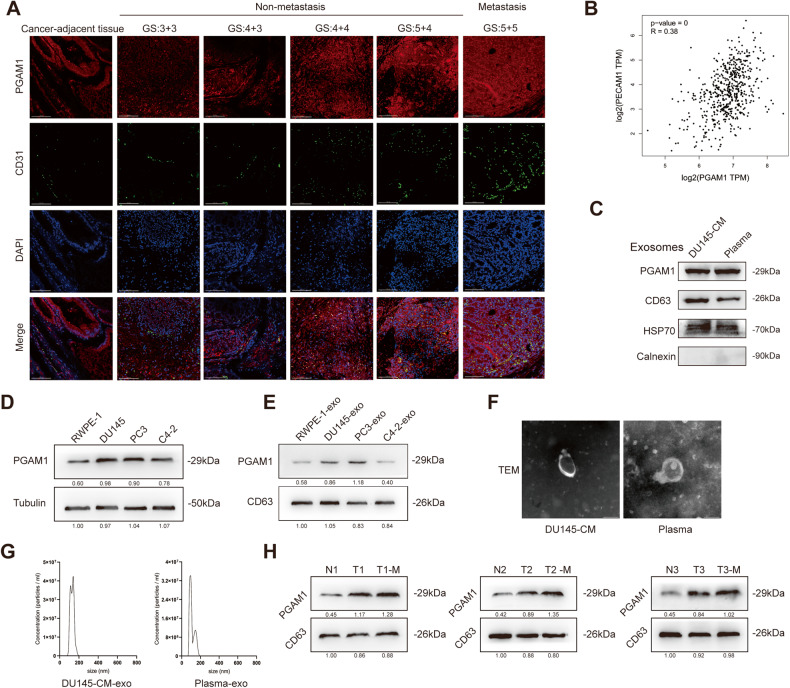
The exosome PGAM1 is involved in the metastasis of PCa. **A** IF analysis of PCa tissues validated the expression of PGAM1 and CD31 in PCa tissues (GS indicates Gleason grade group). The scale bar represents 100 μm. **B** The expression correlation between PGAM1 and CD31 was inferred by bioinformatics (GEPIA 2 (cancer-pku.cn)). **C** Western blotting analysis of PGAM1, CD63, HSP70, Calnexin in exosomes from DU145-CM (cell medium) and PCa plasma. **D**, **E** Western blotting analysis of PGAM1 in RWPE-1, PCa cell lines (DU145, PC-3, C4-2) and their exosomes. **F** Transmission electron microscopy of exosomes derived from DU145-CM and PCa plasma. The scale bar represents 100 nm. **G** Nanoparticle tracer analysis exhibits the size and distribution of exosomes isolated from DU145-CM and PCa plasma. **H** Detection of PGAM1 in plasma exosomes of normal persons and PCa patients by western blot. (N represents normal persons, T represents PCa patients, T-M represents PCa patients with metastasis).

We have previously demonstrated that high PGAM1 expression enhanced the proliferative capacity and attenuated apoptosis of cells in PCa [24]. To determine the involvement of exosomal PGAM1 in PCa cell metastasis, we performed immunofluorescence (IF) staining on clinical samples. The results revealed a significant increase in PGAM1 and CD31 (vascular endothelial cell marker) expression in tumor tissues of patients with PCa compared to adjacent tissues. In addition, a positive correlation was observed between high PGAM1 as well as CD31 expression and high Gleason scores. Interestingly, a significant increase in PGAM1 and CD31 expression was observed in patients with metastatic PCa compared to patients with non-metastatic PCa (Fig. 1A). Next, we identified a strong positive relationship between PGAM1 and CD31 using bioinformatics (Fig. 1B). Finally, we performed immunohistochemistry (IHC) to validate PGAM1 expression in tumors and adjacent tissues in patients with PCa. IHC and IF results were similar. In addition, high PGAM1 expression was observed in patients with metastatic PCa compared to patients with non-metastatic PCa (Supplementary Figs. 1D and 1E).

Next, we determined PGAM1 expression in PCa cells and normal immortalized prostate epithelial cells. The results demonstrated an increase in PGAM1 expression in DU145, PC3 and C4-2 cells compared to RWPE-1 (Fig. 1D). To determine PGAM1 levels in exosomes, we isolated exosomes from DU145 cells supernatant and plasma of patients with PCa using ultracentrifugation. Western blotting (WB) showed that PGAM1, CD63 (exosome marker), and HSP70 were expressed by the isolated exosomes. Calnexin was barely detectable in the exosomes, indicating that the extracted exosomes were of high purity and not contaminated by other organelles or impurities (Fig. 1C). Next, the exosomes were purified, and transmission electron microscopy (TEM), as well as nanoparticle tracking analysis, were performed on these exosomes (Fig. 1F). The results revealed that exosomes had lipid bilayer membranes, and the average diameter was 40–150 nm (Fig. 1G). DU145 and PC3 cells secreted significantly higher levels of exosomal PGAM1 compared to RWPE-1 (Fig. 1E). To determine and quantify the levels of circulating exosomal PGAM1, we isolated exosomes from the plasma of patients with PCa and healthy controls. A significant increase in exosomal PGAM1 levels was observed in the plasma of patients with PCa, specifically metastatic PCa, compared to healthy controls (Fig. 1H). These results revealed a strong correlation between exosomal PGAM1 and PCa cell metastasis (Supplementary Figs. 1A–C).

### PCa-derived exosomal PGAM1 is taken up by HUVECs

**Fig. 2:**
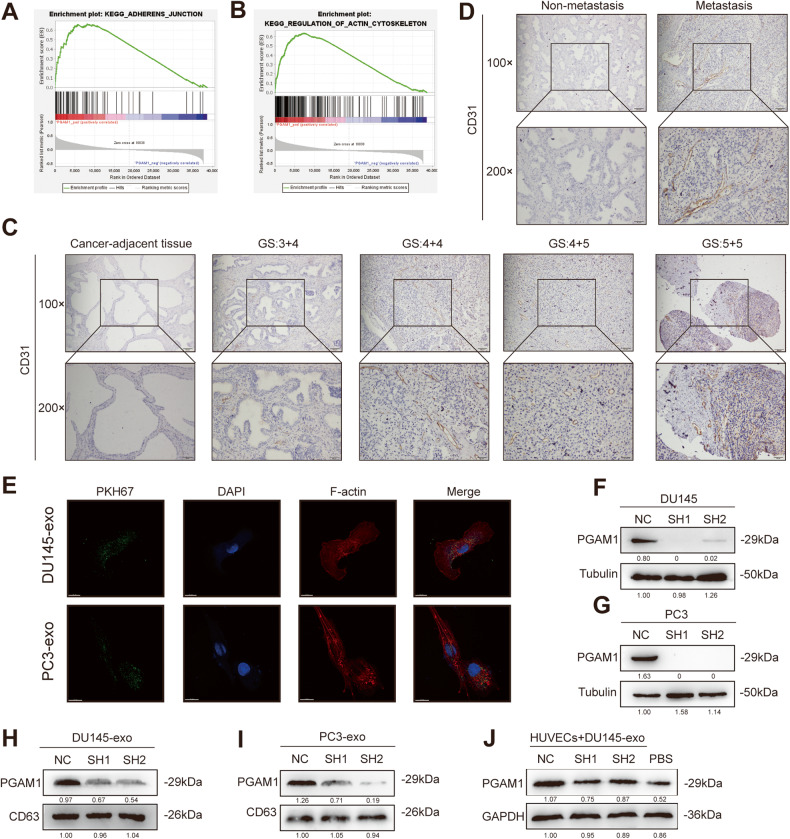
PCa exosome PGAM1 is taken up by HUVECs. **A**, **B** GSEA analysis exhibited that high PGAM1 expression correlated with Cell Adhesion (NES = 2.23 > 1, FDR-q = 0) and Actin Cytoskeleton (NES = 2.32 > 1, FDR-q = 0) Signaling pathway Gene Signature. **C** Representative IHC images of CD31 staining in PCa patients and adjacent tissue to the cancer (GS indicates Gleason grade group). **D** Representative IHC images of CD31 staining in PCa patients with or without metastasis. The scale bar in 100× images represents 100 µm. The scale bar in 200× images represents 50 µm. **E** PKH67-labeled exosomes of PCa cell lines co-cultured with HUVECs (DAPI blue stained nuclei, PKH67 green stained exosomes, F-actin red stained Cytoskeletons). The scale bar represents 15 μm. **F**, **G** Transfection efficiency of two PCa lines (DU145, PC3) was measured by western blot. NC negative control, SH1 shPGAM1-1, SH2 shPGAM1-2. **H**, **I** Western blotting analysis of PGAM1 in exosomes from DU145-CM and PC3-CM. **J** Western blotting analysis of PGAM1 in HUVECs after co-culture with exosomes secreted by DU145-CM. Controls were treated with an equal volume of PBS.

We obtained data on PGAM1 from the Cancer Genome Atlas and performed gene set enrichment analysis on PGAM1 regulatory gene signatures. The results revealed that PGAM1 activated the actin-cytoskeleton and the cell adhesion junction-related signaling pathways (Fig. 2A, B). The cytoskeleton, specifically the actin family of proteins, mediates the changes in the cell shapes and is significantly involved in various stages of angiogenesis [25, 26]. In addition, IHC results revealed a significant increase in CD31 expression in tumor tissues of patients with metastatic PCa compared to patients with non-metastatic PCa (Fig. 2C, D).

HUVECs were cultured with exosomes derived from PKH67-labeled DU145 and PC3 cells. The results revealed that PCa cell-derived exosomes were taken up by HUVECs (Fig. 2E). Next, we successfully constructed stable *PGAM1* knockdown DU145 and PC3 cells (Fig. 2F, G). The exosomes derived from *PGAM1* knockdown DU145 and PC3 cells were called DU145-SH-PGAM1-EXO and PC3-SH-PGAM1-EXO, respectively. The exosomes derived from DU145 and PC3 cells in the *PGAM1* knockdown-negative control group were referred to as DU145-NC-PGAM1-EXO and PC3-NC-PGAM1-EXO, respectively. Next, we determined the PGAM1 expression pattern in exosomes. A significant decrease in PGAM1 expression was observed in exosomes derived from *PGAM1* knockdown DU145 and PC3 cells (Fig. 2H, I). This indicated that PGAM1 expression in PCa cells-derived exosomes positively correlated with changes in PGAM1 expression in cells. Compared to PC3 cells, DU145 cells have higher metastatic, invasive, and angiogenic abilities [27]. Therefore, DU145 cells were used for subsequent experiments. Next, we cultured HUVECs with DU145-NC-PGAM1-EXO, DU145-SH-PGAM1-EXO, and phosphate-buffered saline (PBS) individually. A significant increase in PGAM1 expression was observed in HUVECs treated with DU145-NC-PGAM1-EXO compared to DU145-SH-PGAM1-EXO and PBS (Fig. 2J). Together, PKH67 uptake results indicate that PGAM1 from PCa cells was transferred to HUVECs by exosomes.

### PCa-derived exosomal PGAM1 promotes angiogenesis, migration, and proliferation of HUVECs

**Fig. 3:**
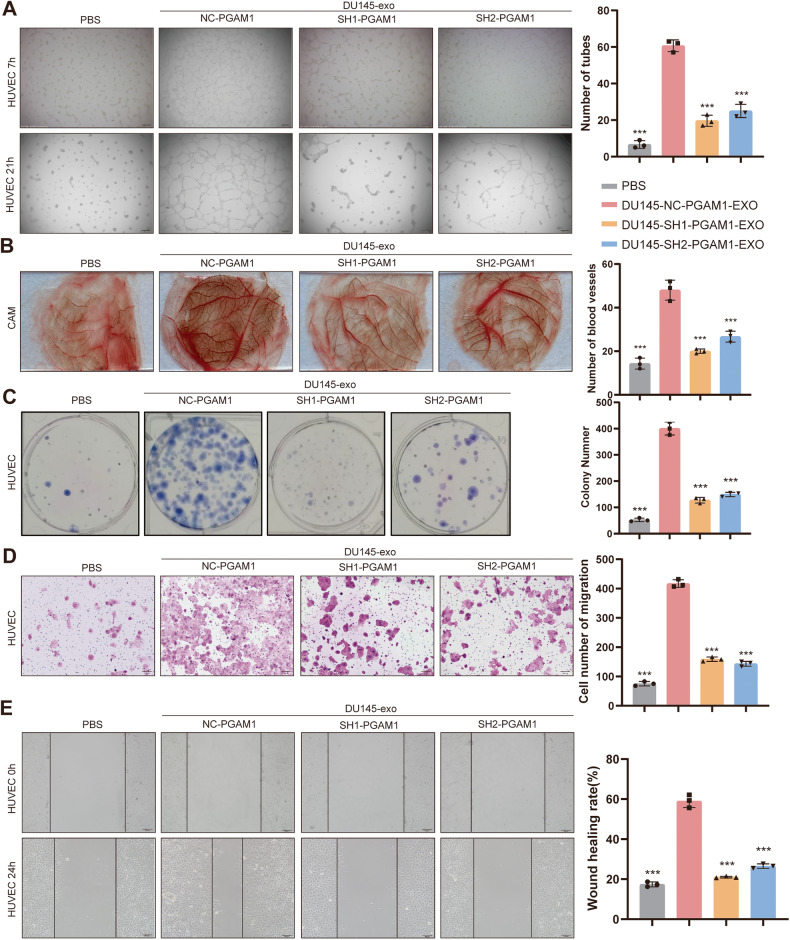
PCa exosome PGAM1 promotes angiogenesis in HUVECs in vitro. HUVECs were treated with exosomes isolated from the DU145-CM for 24 h prior to the following assays. Controls were treated with an equal volume of PBS. **A**, **B** An in vitro Matrigel tube formation assay and CAM assays was performed to assess the angiogenic capacity of HUVECs. And The number of branches per highpower field was analyzed. The scale bar represents 200 μm. **C** Colony-formation assays were performed to assess the proliferative capacity of HUVECs. **D** Transwell assay was performed to evaluate the invasive ability of HUVECs. The scale bar represents 200 μm. **E** Wound healing assays were performed to assess the migration ability of HUVECs. The scale bar represents 100 μm. (Error bars represent means ± SD; ***P < 0.001).

We cultured HUVECs with PCa-derived exosomal PGAM1 to determine the functions of exosomal PGAM1 in HUVECs. Our results revealed high exosomal PGAM1 protein expression in the DU145-NC-PGAM1-EXO group compared to the DU145-SH-PGAM1-EXO group at the same concentration. Matrigel tube formation and chicken chorioallantoic membrane (CAM) assay results revealed that HUVECs treated with DU145-NC-PGAM1-EXO had enhanced angiogenic activity compared to HUVECs treated with DU145-SH-PGAM1-EXO and PBS (Fig. 3A, B). Colony formation assay showed a significant increase in the proliferative ability of HUVECs treated DU145-NC-PGAM1-EXO compared to HUVECs treated DU145-SH-PGAM1-EXO and PBS (Fig. 3C). Transwell and the wound healing assays revealed an increase in the migratory and motility of HUVECs in the DU145-NC-PGAM1-EXO group compared to the DU145-SH-PGAM1-EXO and PBS groups (Fig. 3D, E). These results indicate that the PCa exosomal PGAM1 promotes angiogenesis in HUVECs.

### PGAM1 interacts with ACTG1 in HUVECs

**Fig. 4:**
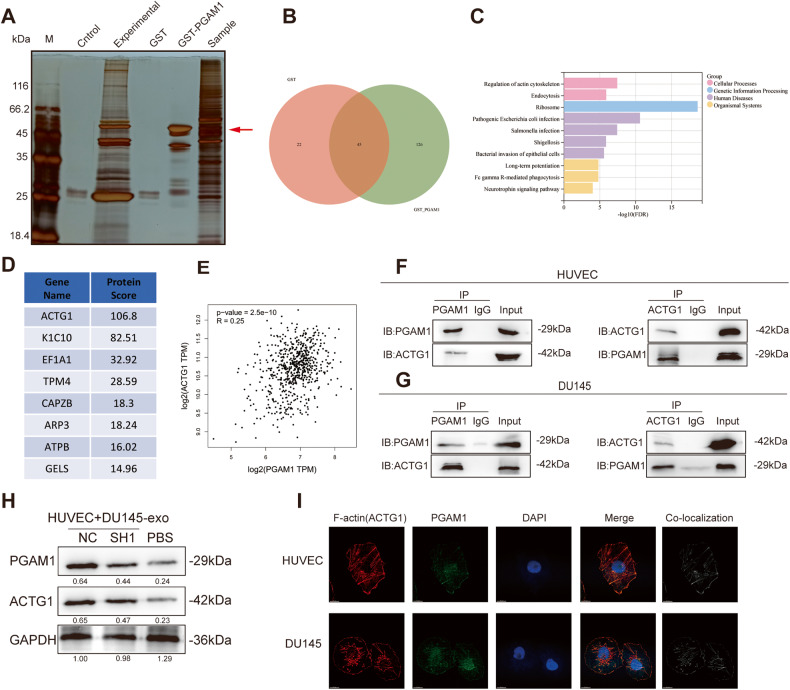
PGAM1 and ACTG1 bind in HUVECs. **A** GST-pulldown enriched protein silver staining assay. **B** Venn diagram of GST and GST-PGAM1 differential proteins. **C** GSEA pathway enrichment map of GST-PGAM1 pull-down proteins. **D** Scoring ranking of GST-PGAM1 pull-down proteins. **E** The expression correlation between PGAM1 and ACTG1 was inferred by bioinformatics. (GEPIA 2 (cancer-pku.cn)). **F**, **G** Co-IP and western blotting assays demonstrated the binding of PGAM1 and ACTG1 in HUVECs and DU145 cells without any intervention. **H** Western blotting analysis of PGAM1 and ACTG1 in HUVECs after co-culture with exosomes secreted by DU145-CM Controls were treated with an equal volume of PBS. **I** IF was used to analyze the co-localization of PGAM1 (green), ACTG1 (red) and DAPI (blue) in HUVECs and DU145 cells. Co-localization indicates the co-localization analysis of PGAM1 and ACTG1 within HUVECs and PCa cells. The scale bar represents 15 μm.

Next, we determined the underlying mechanism of PCa-derived exosomal PGAM1 in promoting angiogenesis in HUVECs. Therefore, we screened for downstream interacting proteins using the Glutathione-S-transferase (GST)-pulldown assay. The results revealed there were many specific binding protein bands in the experimental group relative to those of the control group, as evidenced by silver staining (Fig. 4A). Next, two proteins screened using GST-pulldown assay were identified using mass spectrometry (MS). The total number of proteins identified in the GST and GST-PGAM1 samples were 67 and 171, respectively (Fig. 4B). Of 171 proteins in GST-PGAM1 samples, we subsequently analyzed 126 proteins and excluded two overlapping proteins. Next, we analyzed the pathways enriched by these proteins, and the results revealed that PGAM1-related proteins were enriched in the energy metabolism-related pathways and biological processes, such as actin cytoskeleton regulation (Fig. 4C). Actin is involved in various cellular activities like cell motility, maintenance of the cytoskeleton, etc. [28]. The protein score was determined using the “ProteinPilot software,” and the proteins identified using MS were ranked (Fig. 4D). ACTG1 was the top-ranked gene. Next, a positive correlation was observed between PGAM1 and ACTG1 expression using the "Gene Expression Profiling Interactive Analysis 2” (GEPIA2) database (Fig. 4E).

Furthermore, in the Co-IP assay. We determined endogenous PGAM1 and ACTG1 expression in an immunoprecipitated fraction of HUVECs using an anti-PGAM1 antibody. Next, we immunoprecipitated ACTG1 in cells using an anti-ACTG1 antibody, and the results revealed a significant decrease in PGAM1 expression in cells (Fig. 4F), and consistent results were obtained in DU145 cells (Fig. 4G). To explore the specific interaction sites of PGAM1 and ACTG1, we first predicted the interaction amino acid sites of PGAM1 and ACTG1 through the HADDOCK server. The results showed possible interactions between MET-1, GLU-2, GLU-3, TYR-91, GLU-99 of PGAM1 and ASN-223, LYS-222, LYS-176, ARG-180, LYS-5 of ACTG1 (Fig. S2A). We then constructed plasmids expressing flag-tagged PGAM1 full-length, truncated amino acid 1-9 or amino acid 91-99 sequences and performed flag-IP immunoprecipitation experiments. As a result, only PGAM1 with amino acids 91-99 deleted (PGAM1^Δ91-99^) could not interact with ACTG1, and amino acids 91-99 of PGAM1 were tentatively identified as its specific site of interaction with ACTG1 (Fig. S2B). Subsequently, Transwell or Matrigel tube formation assays show that PGAM1^Δ91-99^ could not rescue the invasion of DU145 or angiogenesis ability of HUVEC due to endogenous knockdown of PGAM1 (Fig. S2C–F).

We treated HUVECs with DU145-NC-PGAM1-EXO, DU145-SH1-PGAM1-EXO, and PBS separately and determined ACTG1 and PGAM1 expression. PGAM1 and ACTG1 expression in the DU145-NC-PGAM1-EXO-treated HUVECs were significantly higher compared to the DU145-SH1-PGAM1-EXO- and PBS-treated HUVECs (Fig. 4H). Next, we performed an IF staining to determine the localization of PGAM1 and ACTG1 proteins in cells. *ACTG1* encodes for an isoform of the actin family of proteins. Actin changes its form from globular monomers (G-actin) to active fibrillar chains (F-actin) to mediate pathophysiological activities in cells. Thus, active F-actin performs the motor and migratory functions [29]; hence, fluorescent phalloidin, a specific dye for F-actin, was used to determine actin localization and PGAM1 expression. PGAM1 and ACTG1 were co-localized in the cytoplasm of HUVECs and DU145 cells (Fig. 4I). These results indicated that PCa-derived exosomal PGAM1 was taken up by HUVECs and could interact with ACTG1 to exert its biological functions.

### Exosomal PGAM1 interacts with ACTG1 to induce podosome formation in HUVECs

**Fig. 5:**
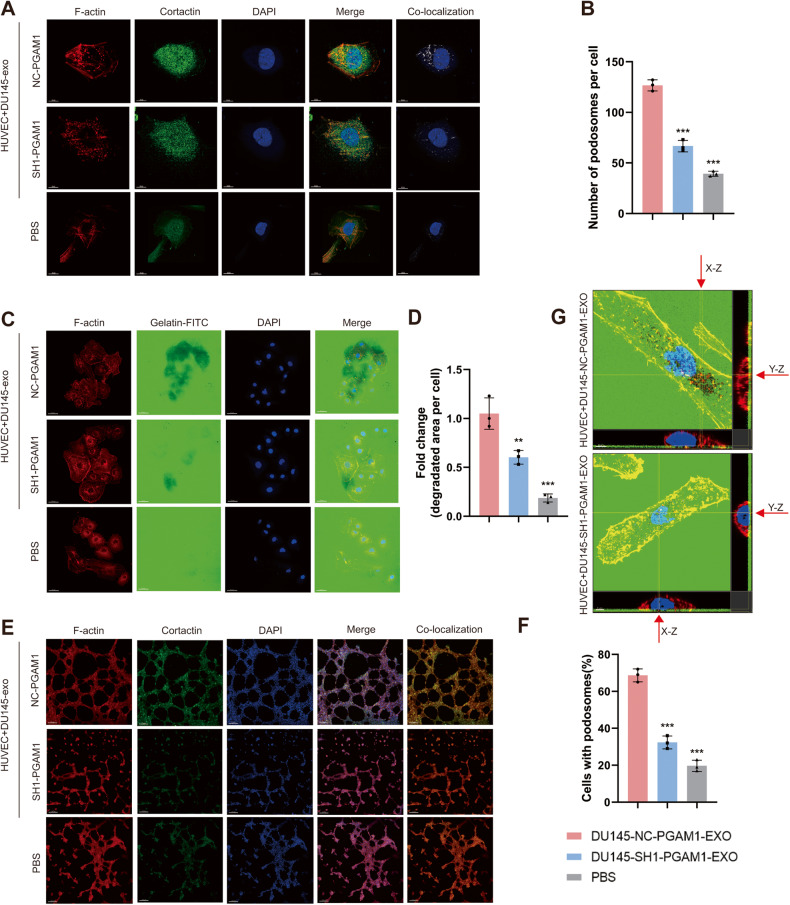
The PCa exosome PGAM1 promotes the production of podosomes in HUVECs. HUVECs were treated with exosomes isolated from the DU145-CM before to the following assays. Controls were treated with an equal volume of PBS. **A** Co-localization of F-actin (red), Cortactin (green) and DAPI (blue) was detected by IF in HUVECs to indicate podosomes. The scale bar represents 10 μm. **B** Quantification of podosomes in HUVECs. **C** HUVECs were plated on FITC-gelatin (green) and stained for F-actin (red) and DAPI (blue). The area of gelatin degradation is shown as a black area below the cells. The scale bar represents 50 μm. **D** Quantification of FITC-gelatin degradation in DU145 cells. **E** F-actin (red), Cortactin (green) and DAPI (blue) staining was used to perform in vitro Matrigel tube formation assays. The scale bar represents 150 μm. **F** Quantification of podosomes in HUVECs. **G** 3D z-stack acquisition was performed in the gelatin degradation experiment, and orthogonal views of the x-z plane and y-z plane are given. The red arrow indicates the area of gelatin degradation. The scale bar represents 5 μm. (Error bars represent means ± SD; **P < 0.01, ***P < 0.001).

Next, we determined if exosomal PGAM1 interacts with ACTG1 to induce podosome formation in HUVECs and is involved in neovascularization sprouting. We performed IF using F-actin and CORTACTIN to identify podosomes in HUVECs [30, 31]. The results revealed a significant increase in the number of podosomes formed by HUVECs in the DU145-NC-PGAM1-EXO group compared to the DU145-SH1-PGAM1-EXO group, while PBS treated group had the least number of podosomes (Fig. 5A, B). Next, we performed a gelatin degradation assay to determine if exosomal PGAM1 affects the ability of HUVECs to degrade the substrate and neovascularization sprouting. We seeded HUVECs on fluorescein (FITC)-conjugated gelatin substrate and the cells were treated based on the specified treatment groups. The area of gelatin degraded by cells indicated the ability of cells to break down the basement membrane. Consistent with the podosome formation results, the area of gelatin degraded by HUVECs in the DU145-NC-PGAM1-EXO group was significantly more compared to the DU145-SH1-PGAM1-EXO group and PBS group (Fig. 5C, D). In the in Matrigel tube formation assay, we observed the formation of podosomes by HUVECs based on IF staining. We observed a significant increase in podosome formation and in tube formation ability of HUVECs treated with exosomal PGAM1 (Fig. 5E, F). Next, we assessed the z-stack projection images of F-actin and gelatin degradation colocalization (Fig. 5G). PGAM1 was colocalized with F-actin and CORTACTIN in the active podosomes of HUVECs. Therefore, the uptake of PCa-derived exosomal PGAM1 by HUVECs promotes neoangiogenesis.

### Knockdown of PGAM1 reduces invadopodia formation by PCa cells

**Fig. 6:**
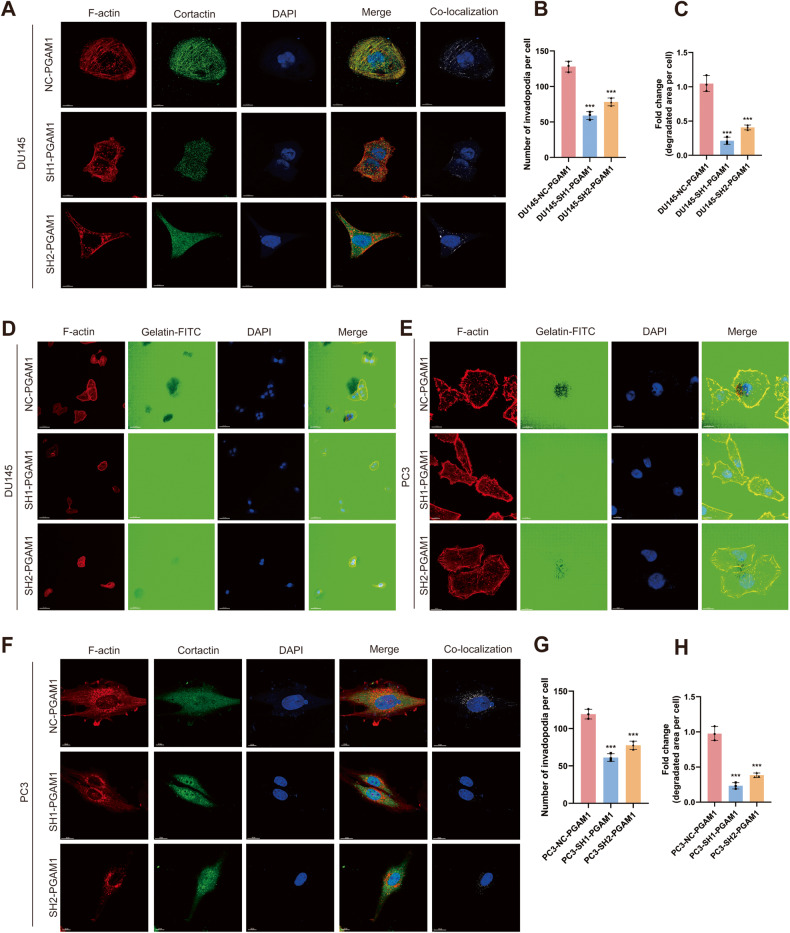
Reduced invadopodia formation in PCa cells with knockdown of PGAM1. **A** Co-localization of F-actin (red), Cortactin (green) and DAPI (blue) was detected by IF in DU145 cells to indicate invadopodia. The scale bar represents 10 μm. **B** Quantification of invadopodia in DU145 cells. **C** Quantification of FITC-gelatin degradation in DU145 cells. **D** DU145-NC-PGAM1 or DU145-SH1-PGAM1 cells were plated on FITC-gelatin (green) and stained for F-actin (red) and DAPI (blue). The scale bar represents 50 μm. **E** PC3-NC-PGAM1 or PC3-SH1-PGAM1 cells were plated on FITC-gelatin (green) and stained for F-actin (red), Cortactin and DAPI (blue). The scale bar represents 10 μm. **F** Co-localization of F-actin (red), Cortactin (green) and DAPI (blue) was detected by IF in PC3 cells to indicate Invadopodia. The scale bar represents 10 μm. **G** Quantification of invadopodia in PC3 cells. **H** Quantification of FITC-gelatin degradation in PC3 cells. (Error bars represent means ± SD; ***P < 0.001).

In vitro and in vivo studies have shown a close correlation between the ability of cancer cells to form invadopodia and their invasive potential [32]. Various oncogenic factors stimulate invadopodia formation by tumor cells, which aids in degrading the basement membrane and invading the extracellular matrix (ECM) as well as the underlying vascular system, thereby triggering a series of metastatic events [33, 34]. Therefore, we investigated if PGAM1 could induce invadopodia formation, thereby causing metastasis. First, DU145 cells were transfected with sh1-PGAM1 and sh2-PGAM1 to knock down PGAM1 expression stably. DU145 cells were transfected with control PGAM1 (NC-PGAM1) were used for comparison. IF staining revealed a significant reduction in invadopodia formation by DU145 in the SH-PGAM1 group compared to the NC-PGAM1 group (Fig. 6A, B). Next, we seeded PCa cells on FITC-conjugated gelatin to determine the ability of PCa cells to degrade gelatin. The results revealed that the area of gelatin degradation by DU145 in the sh-PGAM1 group was significantly less compared to the NC-PGAM1 group (Fig. 6C, D). These results were consistent with invadopodia formation results. Moreover, similar results were obtained using PC3 cells (Fig. 6E–H). Together, these results suggested that PGAM1 promotes invadopodia formation and PCa metastasis.

### Exosomal PGAM1 promotes angiogenesis and metastasis in vivo

**Fig. 7:**
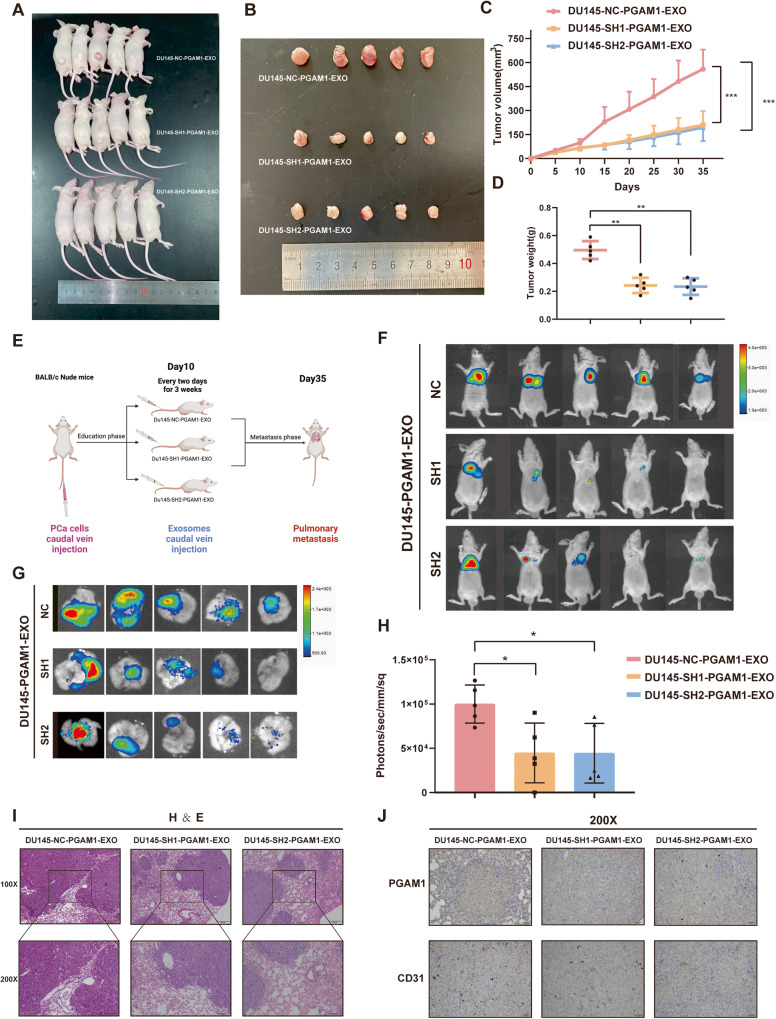
Exosome PGAM1 promotes PCa angiogenesis and metastasis in vivo. **A**, **B** subcutaneous tumor specimen maps. **C** Tumor growth curves were plotted for nude mouse xenografts treated with different groups of exosomes (DU145-NC-PGAM1-EXO, DU145-SH1-PGAM1-EXO and DU145-SH2-PGAM1-EXO) given. **D** The dissected subcutaneous tumor xenografts were weighed and analyzed by One-way analysis of variance (ANOVA). **E** Schematic diagram of caudal vein injection in nude mice with different groups of exosomes. **F**–**H** The luciferase activity (radiance values) of lung metastases at 6 weeks was measured by an in vivo imaging system. **I** Representative images of H&E staining of lung metastatic tumor sections from nude mice. **J** Representative images of PGAM1 and CD31 from IHC stained nude mice with metastatic lung tumors. The scale bar in 100× images represents 100 µm. The scale bar in 200× images represents 50 µm. (Error bars represent means ± SD; *P < 0.05, **P < 0.01, ***P < 0.001).

To determine the effect of PCa-derived exosomal PGAM1 on angiogenesis in vivo, we first constructed subcutaneous xenografted tumors in nude mice using DU145 cells stably expressing luciferase (DU145-luc) (Fig. 7A). Mice were administered PCa-derived exosomes on day 10, followed by every two days for two weeks. The ventral xenografts of nude mice were monitored for 37 days. The results revealed a significant inhibition in tumor growth (Fig. 7B) and a decrease in tumor volume and weight in mice in PGAM1 knockdown group compared to the control group (Fig. 7C, D). Next, we injected DU145-luc in male nude mice via the tail vein to determine the correlation between PCa-derived exosomal PGAM1 and cancer metastasis. We injected PCa-derived exosomes intravenously via the tail vein after day 10, followed by injections once every two days for three weeks (Fig. 7E). Finally, we performed bioluminescence imaging. The results revealed a significant reduction in the bioluminescence signal of lung metastases in mice in the DU145-SH1-PGAM1-EXO, DU145-SH2-PGAM1-EXO group compared to the control DU145-NC-PGAM1-EXO group (Fig. 7F–H). We also performed histological analysis to verify lung metastases (Fig. 7I). IHC results revealed a significant reduction in PGAM1 and CD31 expression in metastatic lung tumors of mice in the DU145-SH1-PGAM1-EXO, DU145-SH2-PGAM1-EXO group compared to the control DU145-NC-PGAM1-EXO group (Fig. 7J). These results indicate that PCa-derived exosomal PGAM1 promotes angiogenesis and metastasis in PCa.

## Discussion

**Fig. 8:**
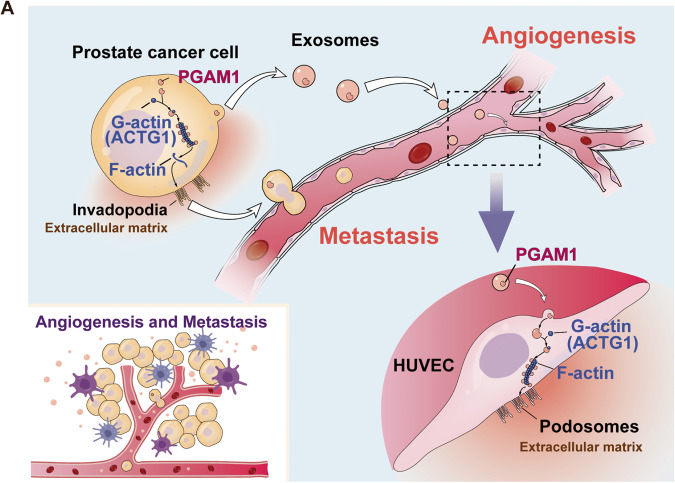
Schematic diagram of the potential mechanism. **A** Schematic representation of the potential molecular mechanism of exosomal PGAM1-induced angiogenesis for PCa metastasis.

Reducing cancer cell invasion and inhibiting angiogenesis are promising therapeutic approaches for treating patients with cancer [35]. Therefore, it is necessary to identify new targets for inhibiting cancer cell migration and anti-angiogenic therapy. In this study, we showed an increase in exosomal PGAM1 levels in the plasma of patients with metastatic PCa and PCa cell culture medium supernatant. Our results revealed that PCa-derived exosomes could be taken up by HUVECs, thereby affecting their function. Interestingly PGAM1 could bind to ACTG1. Moreover, PGAM1 promoted podosome formation and neovascular sprouting in HUVECs. Additionally, PGAM1 increased invadopodia formation in PCa cells, thereby triggering a series of events leading to cancer cell metastasis (Fig. 8A). Together, these results revealed that PGAM1 could be a new anti-angiogenesis target and reduce PCa metastases.

Studies show that metabolic enzymes play a significant role in cancer progression independent of their metabolic activity [36–38]. PKM2 binds to β-linked protein to enhance the transactivation of HIF-1-targeted genes and promote cell cycle protein D1 expression. Similarly, HK2 regulates cancer cell apoptosis by interacting with the AKT signaling pathway [36–38]. Studies have shown that the glycolytic enzymatic activity of PGAM1 plays a role in cell proliferation, whereas the non-metabolic activity of PGAM1 plays a role in the migratory and invasive abilities of cancer cells [18, 39]. Therefore, in this study, we elucidated the non-metabolic functions of PGAM1 in PCa. Moreover, a study has shown that PGAM1 interacts with ACTA2 (unrelated to its metabolic activity) to promote actin filaments assembly, cancer cell migration, and cell motility [39]. Previous studies have shown that PGAM1 promotes cancer progression by inducing angiogenesis [40, 41]. Therefore, our results revealed an in-depth and comprehensive mode of action of PGAM1 in cancers. Additionally, PGAM1 could serve as an important diagnostic and therapeutic target for treating patients with cancer.

Tumor cells actively produce, secrete, and use exosomes that can alter and reshape the TME, promote the migration and invasion of cancer cells, and induce angiogenesis, thereby triggering a series of events leading to cancer metastases [42]. Tumor-derived exosomes contain antigens and nucleic acids specific to the tumor and could severe as diagnostic and predictive biological markers for noninvasive analysis [43]. Zhang et al. showed that nasopharyngeal cancer cells-derived HMGB3 (nuclear exosome) could stimulate angiogenesis, thereby causing cancer metastasis [5]. He et al. showed that ovarian cancer cells-derived exosomal miR-205 induces angiogenesis to promote metastases via the PTEN-AKT pathway [44]. Our results revealed an increase in exosomal PGAM1 in the plasma exosomes of patients with PCa, consistent with the Hosseini-Beheshti et al. results, wherein the proteomic and lipidomic analyses revealed PCa cells-derived exosomes contain PGAM1 as one of the proteins [22]. In addition, we identified that exosomal PGAM1 promotes angiogenesis and migration of HUVECs.

Actin is a highly conserved cytoskeletal protein involved in constructing and maintaining the cytoskeleton, cell motility, etc. ACTG1 encodes for actin isoform, which plays an important role in several cancers and could serve as an early biomarker for cancer [28, 45]. Xiao et al. demonstrated that ACTG1 regulates PCa cell metastasis via the MAPK/ERK signaling pathway and could serve as a marker for PCa cell metastasis [46]. Dong et al. showed that ACTG1 could regulate the proliferation and migration of skin cancer cells via the ROCK signaling pathway [47]. Podosomes and invadopodia are made of an actin-rich core and involve the participation of the actin-binding proteins family [48], which specialized cell-matrix contacts with structures degrading the ECM. Podosomes or invadopodia formation is an important step in vascular sprouting and tumor cell extravasation [49]. Seano et al. found podosome rosettes in HUVECs, a feature of neoangiogenesis. They also found them in angiogenesis models and patients with lung cancer [3]. Our results support this, as exosomal PGAM1 interacts with ACTG1 to promote podosome formation and PCa cell angiogenesis. Chen et al. found invadopodia in patients with pancreatic cancer and other cancers, with a close correlation to cancer cell metastasis [50]. Our results revealed a significant increase in invadopodia formation by PCa cells, which promotes cancer metastasis. We hypothesized that PGAM1 interacts with ACTG1 to mediate these effects. Therefore, we used various molecular biology tools to determine this potential binding between PGAM1 and ACTG1. To the best of our knowledge, our results are the first to show that PCa cells-derived exosomal PGAM1 interacts with ACTG1 to promote cancer metastasis by inducing angiogenesis; however, the exact mechanism of action is still unclear. Together, our results show that exosomal PGAM1 promotes PCa cell metastasis by inducing angiogenesis, thereby providing a new theoretical basis and potential molecular markers for the diagnosis and prognosis of patients with PCa.

## Materials and methods

### Patients and clinical samples

We collected ten prostate tissue specimens of healthy control and patients with PCa undergoing treatment at Zhujiang Hospital of Southern Medical University from 2018–2020. The samples were collected after surgery and evaluated by an experienced pathologist. The samples were flash-frozen in liquid nitrogen and preserved at −80 °C. The average age of the patients was 67 years. The clinical TNM staging of patients was determined based on the 8th edition of the American Joint Committee on Cancer and the Grimson score using the 2016 World Health Organization classification of genitourinary tumors. Prior to tissue collection, the study design was approved by the Southern Medical University Institutional Research Ethics Committee. All patients provided written informed consent.

### Cell lines and cell culture

We obtained HUVECs, RWPE-1 cells (immortalized prostate epithelial cells), and PCa cells like C4-2, DU145, and PC-3 from the stem cell bank of the Chinese Academy of Sciences. All cells tested negative for mycoplasma contamination prior to experiments. First, HUVECs and all PCa cells were cultured in Dulbecco’s modified eagle medium (DMEM) containing high sugar (MA0212, Meilunbio, China) and 10% fetal bovine serum (FBS, Hyclone, Utah, USA). Before collecting supernatants of PCa cells for exosome extraction, PCa cells were washed 1–2 times with PBS and incubated in medium containing exosome-free serum (YOBIBIO, U45-852A) for 48 h. Next, we cultured RWPE-1 cells in keratinocyte serum-free medium (KSFM, No. 10450-013, Gibco, MT, USA) supplemented with 5 ng/mL epidermal growth factor (No. 10744-019, Gibco). All cells were cultured at 37 °C and 5% CO_2_.

### Construction of stable *PGAM1* knockdown cells

We used the Gikai Genetics lentiviral vector (GeneChem, Shanghai, China) to knock down PGAM1 expression in PCa cells following the recommendations of the manufacturer. The sequences of short hairpin RNAs (shRNAs) against PGAM1 were sh1: CATCTGGAGGGTCTCTCTCTGAA.

sh2: TTGCGAGTGCTTTGTTTACTA.

PCa cells were transfected with sh-PGAM1, and stable cell lines were generated by culturing cells in a medium with 5 μg/mL puromycin for 10 days. The specificity and efficacy of knockdown were confirmed.

### RNA extraction and quantitative reverse transcriptase-PCR (qRT-PCR)

Total RNA was extracted using the RNAiso Plus reagent (TaKaRa) and PrimeScript RT kit (TaKaRa). Next, RNA was reverse transcribed to generate complementary DNA based on the procedure specified by the manufacturer. We used SYBR Green PCR Premix (TaKaRa) to perform qRT-PCR on the ABI7500 Fast Real-Time RCR System (Applied Biosystems, USA). Next, primers were designed using the PubMed database and synthesized by DynaScience Biotechnology (Guangzhou, China). All experiments were performed in triplicates. The results were normalized using *GAPDH* as an internal control. Finally, we used the 2-ΔΔCt method for the relative quantification of gene expression.

### Western blotting and antibodies

First, proteins from PCa cells and exosomes were extracted using radio Immunoprecipitation Assay (RIPA) lysis buffer (PA112-01, Biomed, Beijing, China) containing a protease inhibitor, a phosphatase inhibitor, and PMSF (A8260/P1260/P0100, Solarbio, Guangzhou, China) following the standard protocol. Next, 20–30 μg proteins were separated on SDS-PAGE and electro-transferred onto PVDF membranes (Millipore, MA, USA). First, the membranes were blocked using 5% fat-free milk in a Tris-buffered solution with 0.1% Tween-20 (TBST) for 1.5 h, followed by incubation with primary antibody at 4 °C overnight. The primary antibodies used were as follows: rabbit anti-β tubulin (ab108342, Abcam), rabbit anti-flag (ab205606, Abcam), rabbit anti-Calnexin (ab133615, Abcam), mouse anti-GAPDH (ab8245, Abcam), rabbit anti-PGAM1 (16126-1-AP, Proteintech), rabbit anti-ACTG1 (ab200046, Abcam), rabbit anti-CD63 (25682- 1-AP, Proteintech), and rabbit anti-HSP70 (10995-1-AP, Proteintech). Next, the membranes were incubated with secondary anti-rabbit or anti-mouse IgG antibodies conjugated with horseradish peroxidase (Cell Signaling Technology, MA, USA) for 1 h. Finally, the bands were observed using an enhanced chemiluminescence kit (Pierce Biotechnology, IL, USA), and the relative intensities were quantified using ImageJ. All experiments were repeated thrice.

### Isolation of exosomes

Exosomes were isolated as described previously [6]. PCa cells were rinsed with PBS thrice and incubated with an exosome-free FBS medium for 48 h before collecting the cell supernatant. Next, the cell supernatant was centrifuged at 500 × *g* for 5 min to separate the cells, and the supernatant was centrifugated again at 2000 × *g* for 10 min to separate any residual cell debris. This supernatant was centrifugated at 10,000 × *g* for 30 min at 4 °C to isolate macromolecular proteins and centrifugated at 100,000 × *g* for 70 min at 4 °C again. The final precipitate was collected as exosomes, dissolved in PBS, resuspended in RNase-free tubes, and stored at -80 °C for TEM, to determine the protein concentration, RNA isolation, and in vitro as well as in vivo studies. Treatment prior to extraction of exosomes from the serum: First, 10 mL whole blood was drawn from patients and collected in EDTA anticoagulation tubes and centrifugated at 2000 × *g* for 10 min at 4 °C. Next, nearly 4 mL plasma fraction (upper yellow liquid) was aspirated, and the supernatant was centrifuged at 3000 × *g* for 20 min at 4 °C. Finally, the supernatant was collected into a new centrifuge tube and stored at −80 °C or centrifuged at 10,000 × *g* and 100,000 × *g* for exosome extraction.

### TEM of exosomes

After exosome extraction, the samples were taken on a copper mesh, and TEM was performed in Building 58 of the Guangdong Academy of Sciences. A suitable field of view was selected for subsequent analysis. The TEM unit was exclusively handled by an expert in charge at the Guangdong Academy of Chinese Medicine, according to the requirements of the unit.

### Exosome uptake assay

For the exosome uptake assay, we labeled exosomes using the PKH67 Green Fluorescent Cell Linker Mini Kit (Fluorescence, Guangzhou) based on guidelines specified by the manufacturer. Briefly, 10 μg exosomes were resuspended in 500 μL PBS and filtered using a 0.22 μm filter. The exosomes were added to 30% confluent HUVECs in a confocal dish and cultured for 24 h. Next, HUVECs were harvested for IF staining. Finally, the nuclei of HUVECs were stained using 4’,6-diamidino-2-phenylindole (DAPI, Invitrogen™, MA, USA).

### Colony formation assay

For the colony formation assay, First, 500 HUVECs/well were cultured in 2 mL media containing 10% FBS in 6-well plates for 2 weeks. Next, 10 μg exosomes resuspended in 100 μL PBS were added to these 6-well plates every 2 days. Finally, these colonies were fixed and stained using 4% paraformaldehyde (PFA) and Giemsa (Beyotime, Nantong, China), respectively. The experiment was repeated thrice with three wells/groups.

### Transwell migration assay

We used a transwell membrane with an 8.0 μm pore size (Corning Costar, USA) to determine cell migration. First, HUVECs were treated with 10 μg exosomes resuspended in 100 μL PBS for 24 hours. Next, 3 × 10^5^ HUVECs in 200 μL serum-free medium were added to the top chamber, 700 μL medium containing 10% FBS was added in the bottom chamber, and the setup was incubated for 36 h. Next, the cells were fixed and stained using 4% PFA and Giemsa (Boster Ltd., Wuhan, China), respectively. Finally, the top surface of the membrane was wiped, and the cells were imaged using inverted microscopy (DP72, Olympus, Tokyo, Japan). Five random fields were chosen, and the cells were counted using the “Image J” software. All experiments were performed in triplicates.

### Wound healing assay

We treated 1 × 10^5^ HUVECs with 10 μg exosomes resuspended in 100 μL PBS for 24 h. Next, we seeded 1 × 10^5^ HUVECs/well in 6-well plates and cultured until 95% confluent. Next, 200 μl sterile tips were used to create a scratch, washed with PBS to remove floating cells, and cultured in a serum-free medium. Finally, the images were captured at 0 and 24 h using an inverted microscope (DP72, Olympus). The wound healing capacity was determined using the “Image J” software, and the formula used was as follows: wound healing capacity (%) = (initial wound area − final wound area)/initial wound area × 100.

### Matrigel tube formation assay

We treated 1 × 10^5^ HUVECs with 10 μg exosomes resuspended in 100 μL PBS for 24 hours. First, the Matrigel matrix was melted, and 50 μL Matrigel was added to all wells of a 96-well plate and incubated (placed flat) at 4 °C for 10 min, followed by 37 °C for 45 min. Next, 100 μl of 3 × 10^5^ HUVECs suspension/well was added to the Matrigel and incubated at 37 °C for 2–8 h. Finally, the tubes formed were observed using an ortho-fluorescence microscope (BX51), and a network of closed-tube structures was captured from three different tubes.

### CAM assay

A day 6 fertilized eggs were selected (Institute of Animal Husbandry, Guangdong Academy of Agricultural Sciences, China) for the CAM assay. First, the eggs were disinfected using 75% ethanol. Then, to expose the CAM, a window of approximately 1 cm diameter was drilled into the eggshell near the end of the air chamber. Next, a 0.5 cm pore-size sterile filter paper was placed on the CAM, and 100 μl of different exosomes, were added. Next, the window was closed using a piece of sterile tape, and the eggs were incubated at 37 °C and a relative humidity of 80–90% for 2–3 days. Finally, CAM was fixed using a fixation solution (methanol: acetone in a ratio of 1:1) for 15 min, harvested, and isolated. The images were captured using a digital camera. The effect of exosomes on angiogenesis was determined by counting the number of secondary and tertiary vessels using a microscope (Olympus DP72).

### Gelatin degradation assay

The experiment was performed in the dark. Briefly, 50 μg/ml poly-lysine solution (Ubon Bio, Guangzhou, China), 0.5% glutaraldehyde solution (Ubon Bio, Guangzhou, China), and Gelatin-488 (M1303-1, Dakowei Bio, Guangzhou, China) diluted in 1× PBS in the ratio of 1:1, and 5 mg/ml of NaBH_4_ (Ubon Bio, Guangzhou, China) solution was added in a confocal dish in sequence. Incubation at each step was for 25 min, and washing was performed thrice with 1× PBS. And we treated 1 × 10^5^ HUVECs with 10 μg exosomes resuspended in 100 μL PBS in a confocal dish for 24 h. PCa cells were treated similarly, however, without exosomal stimulation. Next, the cells were harvested and imaged. The cells were fixed using a tissue fixing solution (Jiayan Bio, Guangzhou, China) for 15 min and permeabilized using 0.5% Triton X-100 (Jiayan Bio, Guangzhou, China) for 20 minutes. The cells were washed after each step with 1× PBS thrice. Next, the cells were blocked using 1% bovine serum albumin (BSA, Youbang Bio, Guangzhou,China) for 60 min and stained using 150 μL phalloidin (Unibio, Guangzhou, China) for 40–60 min at room temperature. Next, the cells were washed with 1× PBS, and a 50 μL anti-quenching DAPI blocker was added. Finally, the cells were viewed under a confocal fluorescence microscope (Carl Zeiss LSM 880 with Airyscan). The areas of degraded gelatin were quantified using the “Image J” software. All experiments were repeated thrice.

### Animal experiments

All protocols involving animals were performed according to the guidelines specified by Zhujiang Hospital, Southern Medical University. We procured 3–4-week-old male BALB/c nude mice from the Guangdong Medical Laboratory Animal Center (Guangzhou, China). All animals were fed under specific pathogen-free (SPF) conditions. Male BALB/c nude mice were subcutaneously injected with nearly 2.3 ×10^6^ DU145 cells transfected with sh-PGAM1 and shNC vector. The size of tumors was measured using vernier calipers every five days. We created a lung metastasis model by injecting 1.5×10^6^ cells into the tail vein of the nude mice. The quantity of exosomes injected subcutaneously into xenografts and tail veins injection of nude mice was 10 μg (dissolved in 50 μl of PBS) [44]. These mice were anesthetized (Caliper Life Science, MA, USA), and D-luciferin was administered intraperitoneally after 35 days. We captured images using the bioluminescence imaging system (in Vivo FX Pro) to assess luciferase signals. Strong fluorescence signals in the lungs of nude mice indicated metastatic tumor sites. The mice were sacrificed, and the lungs were harvested to detect bioluminescence signals. Finally, xenograft tumors and lung metastases were verified by hematoxylin & eosin, and IHC staining.

### Immunohistochemistry (IHC)

We performed IHC to determine protein expression in the prostate tissue using the PV-6000-6.0 kit (Zhongshan Jinqiao Biotechnology, Beijing, China). The primary antibodies used were as follows: PGAM1 (1:500, 16126-1-AP, Proteintech) and CD31 (1:500, ZM-0044, Zhongshan Jinqiao Biotechnology). The experiment was performed as per the guidelines specified by the manufacturer. We determined the intensity of staining and the proportion of positively stained cells. IHC results were analyzed by three independent senior pathologists using a parallel microscope.

### Immunofluorescence (IF)

The confocal dishes were fixed using 4% PFA at room temperature for 10 min, and paraffin-embedded tissue sections were used for antigen repair and other assays. The samples were washed with PBS thrice, permeabilized using 0.5% Triton X-100 for 20 min, and washed again with PBS thrice. The antigenic sites were blocked using 1% BSA for 60 min. The primary antibodies used were as follows: rabbit anti-PGAM1 (1:200, 16126-1-AP, Proteintech), Cortactin (1:200, AF6436-100, Affinity), CD31 (1:200, CL488-66065, Proteintech), Phalloidin-Tetramethylrhodamine Conjugate (23102, AAT Bioquest). The samples were incubated with primary antibodies at 4 °C overnight and rinsed in PBS. Next, the samples were incubated with specified fluorescent secondary antibodies for 1 h (JiaYan Biologicals, Guangzhou, China), and a DAPI blocker was added (JiaYan Biologicals). The samples were imaged using a confocal fluorescence microscope (LSM 880 Carl Zeiss) and analyzed using Imaris (Imaris 8.1).

### GST-pulldown and MS

For GST pull-down experiments, we incubated GST-PGAM1 fusion protein and total protein (0.5 mg each) on ice for 3 h. Next, Glutathione Sepharose 4B resin columns were loaded with the mixture, rinsed using wash buffer, and the proteins were eluted using wash buffer and 15 mM reduced glutathione. The elutes were separated on a 12% SDS-PAGE, transferred onto PVDF membranes (Millipore, MA, USA), and probed using an anti-His antibody (Sigma-Aldrich, Darmstadt, Germany). GST was used as the negative control and was purchased from Wuhan Genecreate (Wuhan, China). All experiments were performed in triplicates. Next, we identified protein spectra using liquid chromatography-MS/MS, and the data were analyzed using the “ProteinPilot” software.

### Co-immunoprecipitation (Co-IP)

The cells were lysed using the RIPA lysis solution (PA112-01, Biomed, Beijing, China), protease and phosphatase inhibitors, and PMSF (A8260/P1260/P0100, Solarbio, Guangzhou, China) on ice for 30 minutes. Then, the lysates were centrifuged at 12,000 × g for 30 min. The protein concentration in the supernatant was determined using BCA Protein Assay Kit (JiaYan Biology Guangzhou, China). Next, the cell lysates in the IP and IgG groups were incubated with the following primary antibodies PGAM1 (16126-1-AP, Proteintech, 0.3 μg/μl), ACTG1 (ab200046, Abcam, 0.4 μg/μl), and IgG (# 2729S, Cell Signaling Technology, 1 μg/μl) at 4 °C overnight. Next, the magnetic beads (Protein A/G, Bimake, B23202) (Flag, Bimake, B26101) were added, mixed, and incubated at 4 °C overnight on a shaker. Then, the beads were rinsed with a 100:1 ratio of PBS: PMSF five times and denatured by heating after adding an IP concentration of the loading buffer. Finally, proteins were verified using WB.

### Plasmid

pcDNA3.1(+) Plasmid were purchased from Invitrogen (Shanghai, China). The coding sequences of PGAM1^FL^-Flag (wild-type full length PGAM1 fusion DYKDDDDK-tag) were in vitro synthesized and then cloned into pcDNA3.1(+) (GenScript Biotech, Nanjing, China). PGAM1 deletions, PGAM1^Δ1-9^-Flag (1-9 aa deleted) and PGAM1^Δ91-99^-Flag (91-99 aa deleted), were constructed using recombinant polymerase chain reaction. Transfection reagent Lipo3000 was purchased from Invitrogen (L3000015) for transfection of the plasmids on the basis of the manufacturer’s suggestion.

### Molecular docking simulation

The protein structure files for PGAM1 (uniprot ID: P18669) and ACTG1 (uniprot ID: P63261) were downloaded from Alphafold (https://alphafold.com/). Prior to docking, de-watering and dechlorination were performed using PyMOL software. The processed protein molecules were predicted for the PGAM1-ACTG1 model using the protein-protein docking online service platform HADDOCK (https://bianca.science.uu.nl/haddock2.4/). The active residues of PGAM1 were set to be predicted by the CPORT (http://alcazar.science.uu.nl/services/CPORT/) server, and the active residues of ACTG1 were set to be full sequence amino acids. Finally, protein-protein docking was performed using the default parameters of the website, and the best model was output as the PGAM1-ACTG1 model obtained by docking. The docking result was processed and polished with PyMOL software.

### Statistical analysis

We used “GraphPad Prism8.03” (CA, USA) for statistically analyzing the data. We used the student’s *t*-test (two-tailed) for comparing data with normal distribution and the Mann–Whitney *U* test for analyzing data with non-normal distribution. One-way analysis of variance was used for analyzing data with continuous variables. A two-sided test with *P* < 0.05 was considered statistically significant.

## Supplementary information

Original Data File

Supplemental Figure1

Supplemental Figure2

checklist

Statement of supplemental material.

Supplemental Material of GST pull-down1

Supplemental Material of GST pull-down2

## Data Availability

All data generated or analyzed during this study are included in this published article.
