# Supplementary material for: Exosomal PGAM1 promotes prostate cancer angiogenesis and metastasis by interacting with ACTG1
Source: Cell Death Dis. 2023 Aug 4;14(8):502. doi: 10.1038/s41419-023-06007-4 (PMC10403531; doi:10.1038/s41419-023-06007-4)

F1(C)

PGAM1

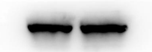

DU145-CM—Plasma

F1(C)

CD63

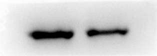

DU145-CM—Plasma

F1(C)

HSP70

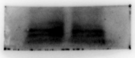

DU145-CM—Plasma

F1(C)

Calnexin

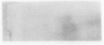

DU145-CM—Plasma

F1(D)

PGAM1

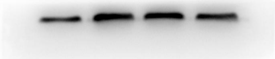

RWPE-1—DU145—PC3—C4-2

F1(D)

Tubulin

RWPE-1—DU145—PC3—C4-2

F1(E)

PGAM1

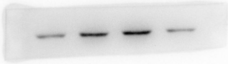

Exosomes
RWPE-1—DU145—PC3—C4-2

F1(E)

CD63

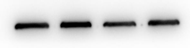

Exosomes
RWPE-1—DU145—PC3—C4-2

F1(H)

PGAM1
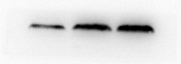

Plasma exosomes
N1—T1—T1-M

F1(H)

CD63
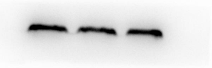

Plasma exosomes
N1—T1—T1-M

F1(H)

PGAM1
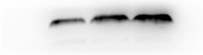

Plasma exosomes
N2—T2—T2-M

F1(H)

CD63
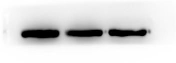

Plasma exosomes
N2—T2—T2-M

F1(H)

PGAM1
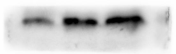

Plasma exosomes
N3—T3—T3-M

F1(H)

CD63
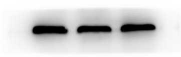

Plasma exosomes
N3—T3—T3-M

F2(F)

PGAM1
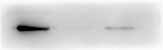

DU145
NC—SH1—SH2

F2(F)

Tubulin
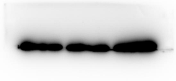

DU145
NC—SH1—SH2

F2(G)

PGAM1
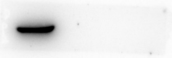

PC3
NC—SH1—SH2

F2(G)

Tubulin
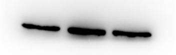

PC3
NC—SH1—SH2

F2(H)

PGAM1

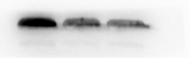

DU145-Exosomes
NC—SH1—SH2

F2(H)

CD63

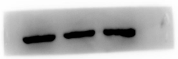

DU145-Exosomes
NC—SH1—SH2

F2(I)

PGAM1

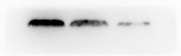

PC3-Exosomes
NC—SH1—SH2

F2(I)

CD63

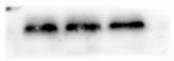

DU145-Exosomes
NC—SH1—SH2

F2(J)

PGAM1

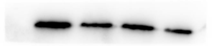

HUVEC+DU145-Exosomes
NC—SH1—SH2—PBS

F2(J)

GAPDH

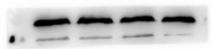

HUVEC+DU145-Exosomes
NC—SH1—SH2—PBS

F4(F)

HUVEC

IP
PGAM1—IgG—Input

IB:PGAM1

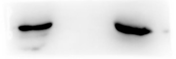

IB:ACTG1

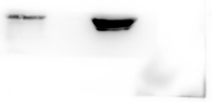

IP
ACTG1—IgG—Input

IB:ACTG1

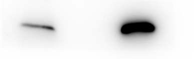

IB:PGAM1

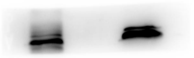

F4(G)

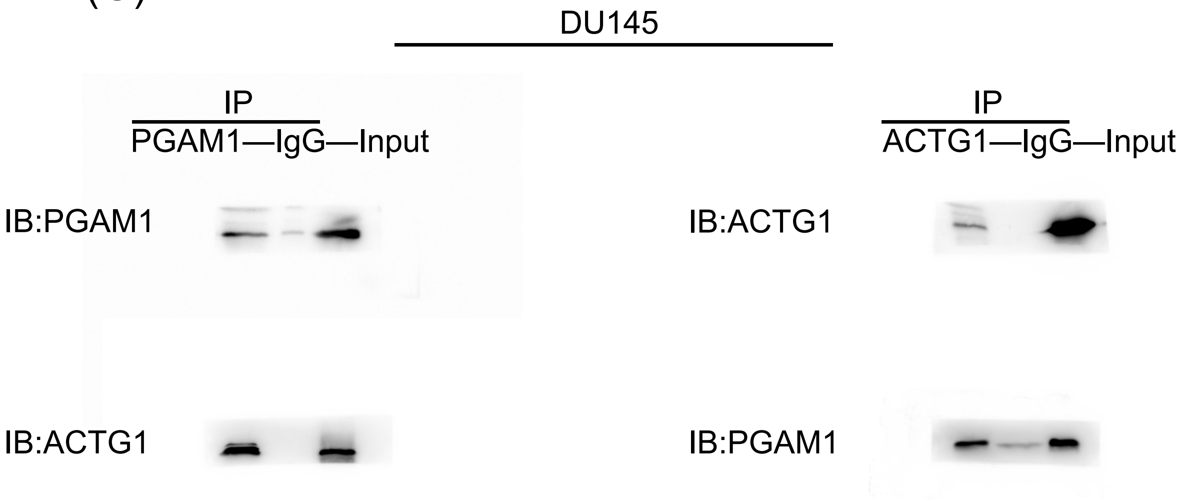

F4(H)

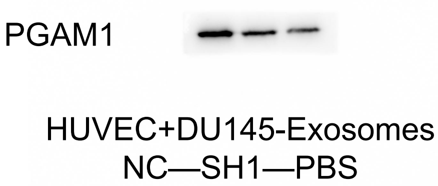

F4(H)

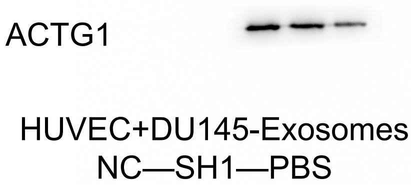

F4(H)

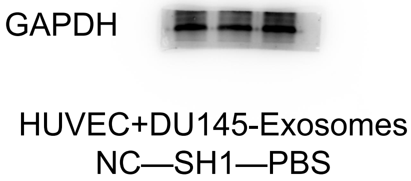

Supplementary F2(B)

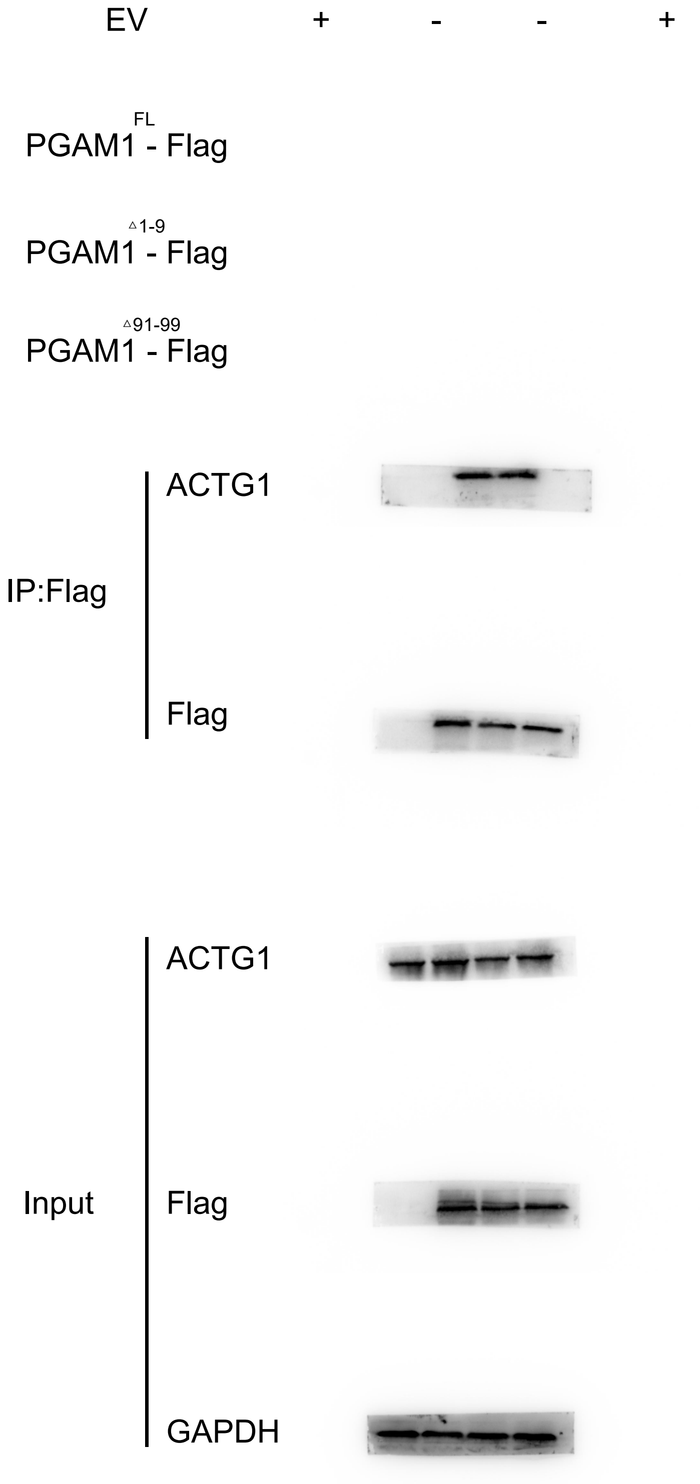

Supplement: Supplementary file 1 — Original Data File [file 41419_2023_6007_MOESM1_ESM.pdf]
