# Supplementary figures and images for: Exosomal PGAM1 promotes prostate cancer angiogenesis and metastasis by interacting with ACTG1
Source: Cell Death Dis. 2023 Aug 4;14(8):502. doi: 10.1038/s41419-023-06007-4 (PMC10403531; doi:10.1038/s41419-023-06007-4)

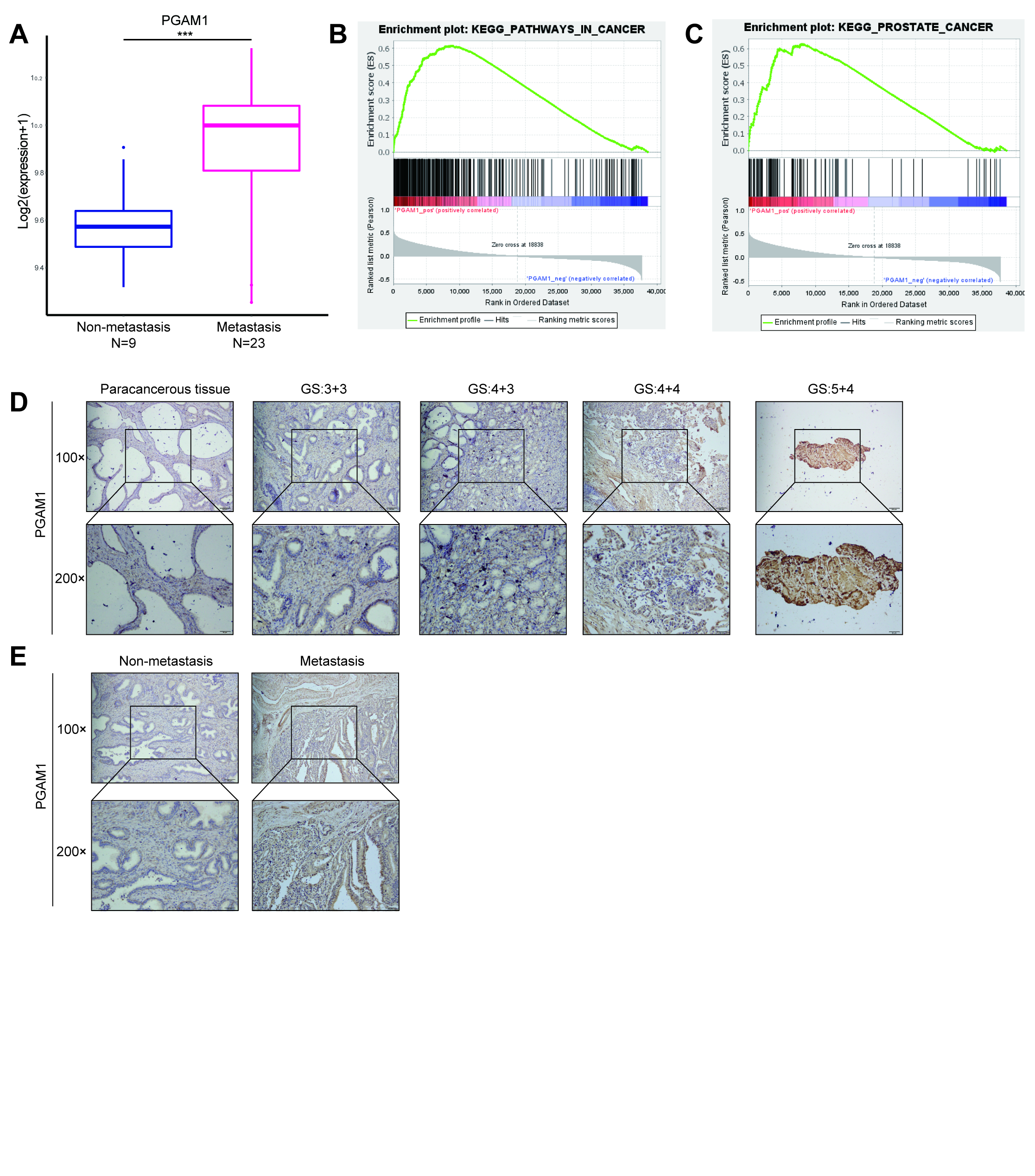

Supplement: Supplementary file 2 — Supplemental Figure1 [file 41419_2023_6007_MOESM2_ESM.tif]

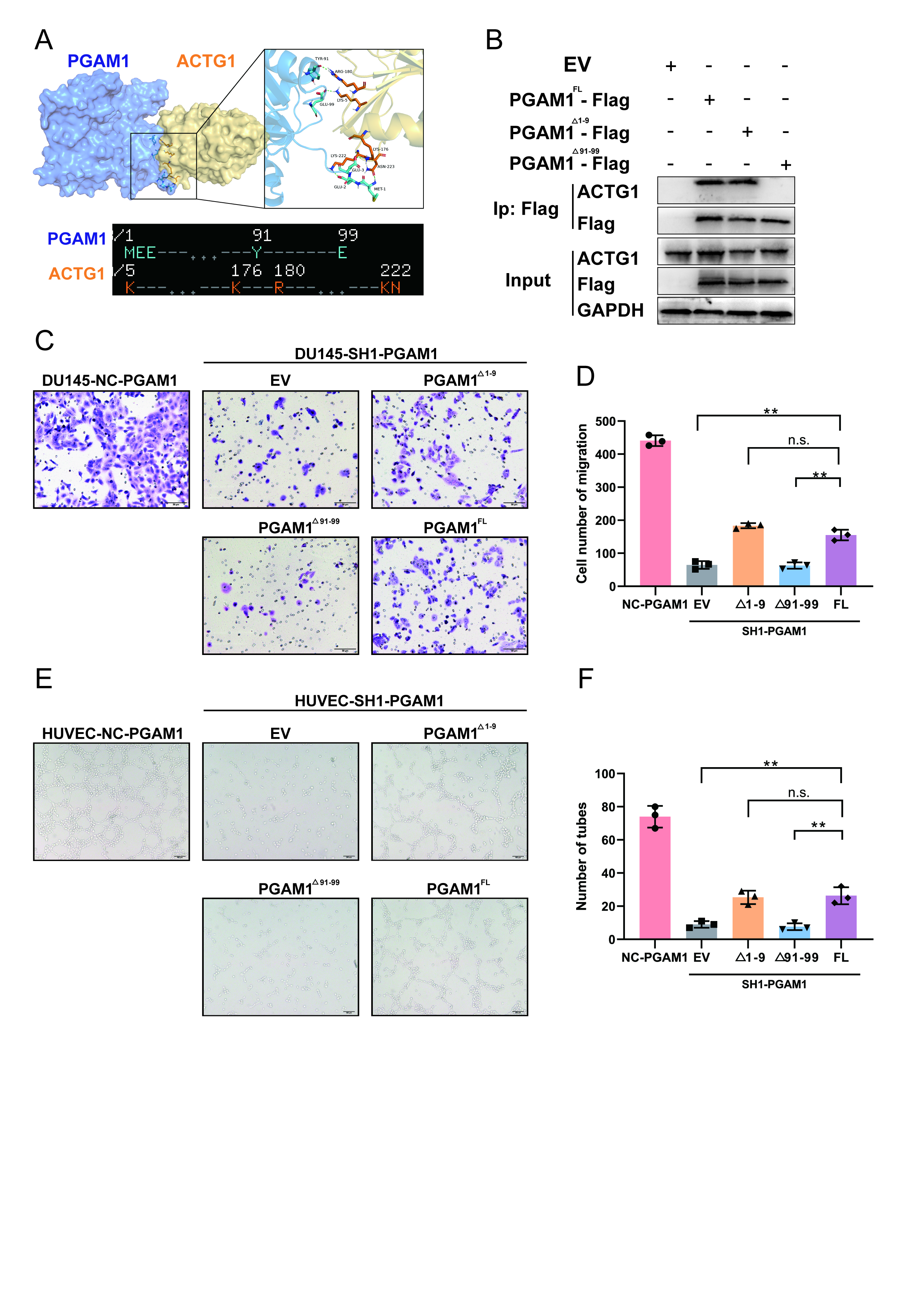

Supplement: Supplementary file 3 — Supplemental Figure2 [file 41419_2023_6007_MOESM3_ESM.tif]
