# Supplementary material for: Exosomal PGAM1 promotes prostate cancer angiogenesis and metastasis by interacting with ACTG1
Source: Cell Death Dis. 2023 Aug 4;14(8):502. doi: 10.1038/s41419-023-06007-4 (PMC10403531; doi:10.1038/s41419-023-06007-4)
Supplement: Supplementary file 5 — Statement of supplemental material. [file 41419_2023_6007_MOESM5_ESM.docx]

**Supplementary Fig. 1 PGAM1 is an important factor in PCa metastasis. A** PGAM1 is highly levels in patients with metastatic PCa in the TCGA database. **B, C** GSEA analysis of PGAM1 high expression correlates with gene signature of PCa signaling pathway (B:NES=2.30≥1, FDR-q=0.0; C: NES=2.14≥1, FDR-q=0.0). **D** Representative IHC images of PGAM1 staining in PCa patients and adjacent tissue to the cancer (GS indicates Gleason grade group). **E** Representative IHC images of PGAM1 staining in PCa patients with or without metastasis. The scale bar in 100x images represents 100 µm. The scale bar in 200x images represents 50 µm.

**Supplementary Fig. 2 Prediction and validation of PGAM1 and ACTG1 interaction sites. A** HADDOCK predicted interactions between MET-1, GLU-2, GLU-3, TYR-91, GLU-99 of PGAM1 and ASN-223, LYS-222, LYS-176, ARG-180, LYS-5 of ACTG1. **B** Immunoprecipitation detection of PGAM1 and its mutant proteins interacting with ACTG1. **C, D** Transwell assays were performed to assess the effect of PGAM1 1-9aa deletion (PGAM1^Δ1-9^) and 91-99aa (PGAM1^Δ91-99^) deletion on the invasive ability of DU145. Scale bars represent 200 μm **E, F** Matrigel tube formation assays were performed to assess the effect of PGAM1 1-9aa deletion and 91-99aa deletion on the migration ability of HUVEC. The scale bar represents 100 μm. (Error bars represent means ± SD; **P < 0.01; n.s., not significant; EV, empty vector).

“GST_PGAM1_protein_identified” contains a list of proteins identified in the GST-PGAM1 samples.

“GST_protein_identified” contains a list of proteins identified in the GST samples.

The Original western blots are deposited in the “original western blots” folder.
